# Challenges in the use of atomistic simulations to predict solubilities of drug-like molecules

**DOI:** 10.12688/f1000research.14960.2

**Published:** 2019-01-04

**Authors:** Guilherme Duarte Ramos Matos, David L. Mobley

**Affiliations:** 1 Department of Chemistry, University of California, Irvine, Irvine, California, USA; 2 Departments of Pharmaceutical Sciences and Chemistry, University of California, Irvine, Irvine, California, USA

**Keywords:** solubility, molecular crystals, free energy calculations, chemical potentials, solvation

## Abstract

**Background: **Solubility is a physical property of high importance to the pharmaceutical industry, the prediction of which for potential drugs has so far been a hard task. We attempted to predict the solubility of acetylsalicylic acid (ASA) by estimating the absolute chemical potentials of its most stable polymorph and of solutions with different concentrations of the drug molecule.

**Methods:** Chemical potentials were estimated from all-atom molecular dynamics simulations.

We used the Einstein molecule method (EMM) to predict the absolute chemical potential of the solid and solvation free energy calculations to predict the excess chemical potentials of the liquid-phase systems.

**Results:** Reliable estimations of the chemical potentials for the solid and for a single ASA molecule using the EMM required an extremely large number of intermediate states for the free energy calculations, meaning that the calculations were extremely demanding computationally. Despite the computational cost, however, the computed value did not agree well with the experimental value, potentially due to limitations with the underlying energy model. Perhaps better values could be obtained with a better energy model; however, it seems likely computational cost may remain a limiting factor for use of this particular approach to solubility estimation.

**Conclusions:** Solubility prediction of drug-like solids remains computationally challenging, and it appears that both the underlying energy model and the computational approach applied may need improvement before the approach is suitable for routine use.

## Introduction

Solubility is a critical property for pharmaceutical drug discovery; problems with solubility can frustrate drug discovery efforts and prevent treatments from working. The bioavailability of a drug depends on the solubility difference between different crystal structures (polymorphs), dose, drug permeability and formulation ^1^, so solubility plays a key role. Solubility problems can be unexpected and can pose crucial obstacles that even threaten the administration of care. For example, a well-documented case occurred in the late 1990s, when ritonavir, an HIV-protease inhibitor marketed as Norvir, failed dissolution requirements ^2^ due to the sudden accidental discovery of an extremely stable new polymorph which actually threatened drug supply ^2^. Thus considerable effort has already been devoted to the methods to predict crystal polymorphs ^3– 9^, but much less attention has been given to methods to predict solubilities, with or without likely polymorphs as input.

The results of a recent solubility challenge ^10, 11^ provide a helpful glimpse into the state of the field. Employed methods were entirely empirical and, though quite diverse (e.g. neural networks ^12^, deep learning ^13^, and quantitative structure-property relationships ^14^), had notable failures. Key limitations included the dependence on the availability of training data for similar compounds ^11^.

Some newer methods attempt to predict solubilities based on a physical description of the interactions in solution and in the solid state, yielding results that are in principle rigorous given an accurate energy model and an adequate method. In these approaches, molecular systems are described using force fields, i.e, potential energy functions that contain parameters describing bonds, atoms, electrostatic and non-electrostatic interactions. Molecular dynamics or Monte Carlo simulations are commonly used to sample different configurations of the system described by an energy model called a force field, allowing estimation of various physical properties. With these techniques, some recent work calculated aqueous solubilities using thermodynamic cycles encompassing the crystal, the ideal gas, and an infinitely dilute solution of a given molecule ^15, 16^. When the structure of the solid is unknown, some studies have substituted simulations of solid melts in place of a structure of the solid ^17– 20^.

While these physical methods for predicting solubilities have received some attention in the literature, most are still in their infancy, with only a handful of studies applying them, and it is not yet clear how broadly applicable they will be ^17– 20^, and others have only been suggested or demonstrated in proof-of-principle tests ^16, 21– 23^.

Our view is that the time is ripe for physical methods to predict solubility, especially given the routine nature of solvation free energy calculations ^24– 29^, which comprise essentially half of the solubility problem (see the Theory section). Polymorph and crystal structure prediction successes also mean that we may often have a suitable crystal structure of the compound as an input ^3– 5, 8, 9, 30– 35^, so what remains is to predict the solubility given a crystal structure and simulations of the relevant phases.

Here, we focus on adapting, testing, extending and generalizing an approach for solubility prediction, with the hope it will eventually see routine use. This method uses all-atom molecular dynamics simulations to estimate absolute chemical potentials and predict aqueous solubilities of molecular solids, given the crystal structure (or an estimate thereof) as input.

While our approach builds on earlier approaches, it does provide several significant advances. First, we are able to compute solubilities for flexible molecules, like acetylsalicylic acid, Second, we employ a revised thermodynamic solubility that enhances and improves the precision of calculations of the solubility of methanol. Third, while our approach is relatively expensive computationally, there is a clear path forward towards reducing computational cost, and already (at least with a sufficiently accurate force field) it could be suitable for applications in industry.

## Theory

### The solubility of a molecular solid is related to the chemical potentials of each phase

Solubility is defined as the maximum concentration of solute that can be dissolved in a selected bulk solvent. Chemical potentials ( *µ*) of the solid-state solute and the solution are by definition equal at the solubility point, when the solution is in equilibrium with the solid.

$$\mu_{solute}^{solid}=\mu_{solute}^{solution}\;(1)$$

Solid particles precipitate in concentrations higher than the solubility point because the solid phase becomes more stable in these conditions. In principle, we can predict at which concentration a molecule precipitates in solution if we calculate the chemical potentials of the components:

$$\mu_{i}={\left(\frac{\partial A}{\partial N_{i}}\right)}_{V,T,N_{j,j\neq i}}={\left(\frac{\partial G}{\partial N_{i}}\right)}_{P,T,N_{j,j\neq i}}\;(2)$$

where *µ*_*i*_ is the chemical potential of component *i*; *A* is the Helmholtz free energy; *G* is the Gibbs free energy; *N*_*j, j≠i*_ is the number of molecules of each component in the mixture; *V* is the volume of the system; *T* its temperature; and *P* its pressure. Calculations from systems under a constant *V* and *T* yield *A*; *G* is obtained from simulations under constant *P* and *T* conditions. In order to estimate the chemical potential of one component in solution and in its molecular solid, however, we need to know the absolute free energy of the system in these states. We calculated absolute free energies using alchemical free energy calculations.

### Using the Einstein Crystal or Einstein Molecule methods provides a way to compute the chemical potential of the solid

One key challenge in this work is the calculation of the chemical potential of the solid. Here we briefly survey the approach used for such calculations.

Chemical potential of solids are equal to their molar absolute free energies. In order to calculate absolute free energies, however, we need to define a reference state for which we know how to calculate the free energy analytically. The Einstein Crystal Method (ECM) ^36^ is a possible reference state in which a solid is represented by a collection of atoms bound to their lattice positions by a harmonic restraint, i.e, a spring-like potential. Despite the possibility of calculating the free energy of an Einstein Crystal analytically from the equations of statistical mechanics, implementing the ECM results in challenges due to lattice movements ^37^. The Einstein Molecule Method (EMM) ^22, 37– 40^ is somewhat easier to implement because fixing the position of one atom in the lattice (easily implemented with many molecular simulation packages) eliminates the issue with lattice motions ^40^.

Either aproach allows calculation of the absolute free energy of the solid. Specifically, the absolute free energy is obtained by adding the free energy of the reference – either the Einstein Crystal or Einstein Molecule reference state – to the free energies of the transformation path between the reference state and the final state, the molecular solid. In ECM, beginning from the restrained and noninteracting state, one turns on the force field terms creating an intermediate state called the “interacting Einstein Crystal” (IEC). The IEC retains harmonic restraints but also includes full force field interactions. From the IEC state, an additional set of calculations turns off the restraints, reaching the molecular solid with a fixed center of mass (SFCM). A final step involves then releasing the center of mass. The EMM approach involves a similar set of free energy calculations, except there is no need to compute the free energy of releasing a fixed atom in the lattice.

Additional details of both approaches are discussed below.

### Alchemical free energy calculations can be used to calculate absolute free energies

The absolute free energy of a system can be determined if we know its partition function ( *Q*), a function that connects microscopic properties of the system with macroscopic thermodynamic quantities. Unfortunately, it is very hard to calculate the absolute free energy of real systems because we don’t know their partition functions. Free energy calculations allow us to bypass this problem, but require at least two states: a reference state whose free energy can be analytically or numerically found, and a final state of interest ^41, 42^. We chose to calculate the free energy difference using alchemical free energy calculations, a method in which we simulate a series of non-physical intermediates between the end states ^43^.

Each intermediate state in the alchemical path is described by a Hamiltonian *ℋ* ( **q**, **p**; *λ*), i.e, the energy of the state as a function of atomic positions ( **q**), momenta ( **p**) and a coupling parameter ( *λ*):

$$ℋ(q,p;\lambda )=f(\lambda ){ℋ}_{initial}(q,p;\lambda )+g(\lambda ){ℋ}_{final}(q,p;\lambda )\;(3)$$

where *ℋ*_*initial*_ and *ℋ*_*final*_ respectively are the Hamiltonians of the initial and the final state; and *f* ( *λ*) and *g*( *λ*) are functions used to mix the Hamiltonians, and are usually set such that *ℋ* = *ℋ*_*initial*_ at *λ* = 0 and *ℋ* = *ℋ*_*final*_ at *λ* = 1.

A variety of different estimators can be used to analyze alchemical free energy calculations, and have different strengths and weaknesses, as well as different data requirements. Here, we employ several different estimators we introduce briefly in the following.

One way to calculate the free energy difference (Δ *A*) between the end states is Thermodynamic Integration (TI) ^44^:

$$\Delta A={\int_{\lambda =0}^{\lambda =1}\langle \frac{\partial ℋ}{\partial \lambda }\rangle }_{\lambda }\text{d}\lambda \;(4)$$

in which a set of discrete *λ* values correspond to states along the alchemical path. 〈〉 means that we are have to calculate the ensemble average of the derivative between the brackets. TI performs as well as more efficient methods if the integrand is smooth, but breaks down if this condition is not satisfied ^45– 47^.

An alternate free energy estimation method computes Δ *A* directly via:

$$\Delta A=-\frac{1}{\beta }\ln{\langle e^{-\beta [{ℋ}_{final}-{ℋ}_{initial}]}\rangle }_{initial}\;(5)$$

where the ensemble average is calculated over the configurations of the initial state, and *β* is the reciprocal of *k*_*B*_ *T*, the product between the Boltzmann constant and the absolute temperature. We call this approach exponential averaging ^48^ (EXP).

Most free energy calculations involve many intermediates associated with the coupling parameter ( *λ*), allowing simulation of intermediate states in between the two end states of interest. The free energy change between the end points of a path defined by *N* intermediates is:

$$\Delta A=\sum_{n=1}^{N-1}\Delta A_{n\to n+1}\;(6)$$

where Δ *A*_*n*→ *n*+1_ is the free energy difference between ( *n*+1)-th and the *n*-th intermediate states. Equation 5 can be used to calculate the free energy difference between each adjacent pair of states and yields the exact result at the limit of very large samples, but it is inefficient for most applications ^43^.

The Bennett acceptance ratio ^49^ (BAR) provides an estimator that is superior for most purposes. It calculates the free energy difference between the *n*-th and the ( *n* + 1)-th states from the following relationship:

$${\langle \frac{1}{1+\frac{N_{n}}{N_{n+1}}e^{\beta \left(\Delta {ℋ}_{n\to n+1}-\Delta A\right)}}\rangle }_{n}={\langle \frac{1}{1+\frac{N_{n+1}}{N_{n}}e^{\beta (\Delta {ℋ}_{n+1\to n}+\Delta A)}}\rangle }_{n+1}\;(7)$$

where *N*_*n*_ and *N*_*n*+1_ are the number of statistically independent samples in states *n* and *n* + 1, respectively, and Δ *ℋ*_*n*→ *n*+1_ = −Δ *ℋ*_*n*+1→ *n*_ are the Hamiltonian differences between *n* and *n* + 1. BAR is more efficient than EXP ^50, 51^ and minimizes the free energy uncertainty ^49^. Multistate Bennett acceptance ratio ^46^ (MBAR) is an extension of BAR that takes in consideration the degree of configuration space overlap between a given state and all other states in the transformation, whereas BAR only uses the information of neighboring states. MBAR and BAR perform similarly when the spacing between the intermediate states is moderate, but MBAR is the most well-performing free energy estimator ^47^.

### The absolute free energy of a solid is calculated using an ideal system as reference

In this work, we seek to predict the solubilities of molecular solids. Part of this problem requires predicting the free energy or chemical potential of the solid. One way this has been attempted in the past is via the Einstein crystal method (ECM), which calculates the absolute free energy of a solid using an Einstein crystal as a reference state. In this method, the crystal lattice is made of atoms restrained to their positions by a harmonic potential; additionally, the center of mass of the system is held fixed ^36^.

**Figure 1.**
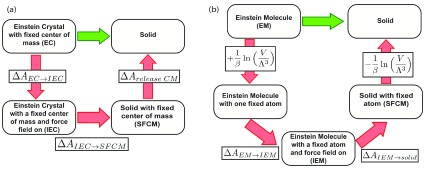
( **a**) Thermodynamic cycle representing the Einstein Crystal Method. ( **b**) Thermodynamic cycle representing the Einstein molecule method (EMM). Note that the EMM requires only two free energy calculations despite being a bigger thermodynamic cycle. The canceling terms in ( **b**) correspond to the free energies of fixing and releasing one atom in the crystal lattice ^37^.

In the ECM, and in this work, the absolute free energy of the molecular solid is found by designing a path where force field terms are progressively turned on, and the harmonic potential position restraints are turned off. The fixed center of mass is important to avoid a quasi-divergence issue when calculating the free energy term of releasing the system from the harmonic position restraints, but the contribution of the fixed center of mass needs to be included in the cycle to obtain the correct absolute free energy for the system ( Figure 1(a)) ^36, 37, 52^.

**Figure 2.**
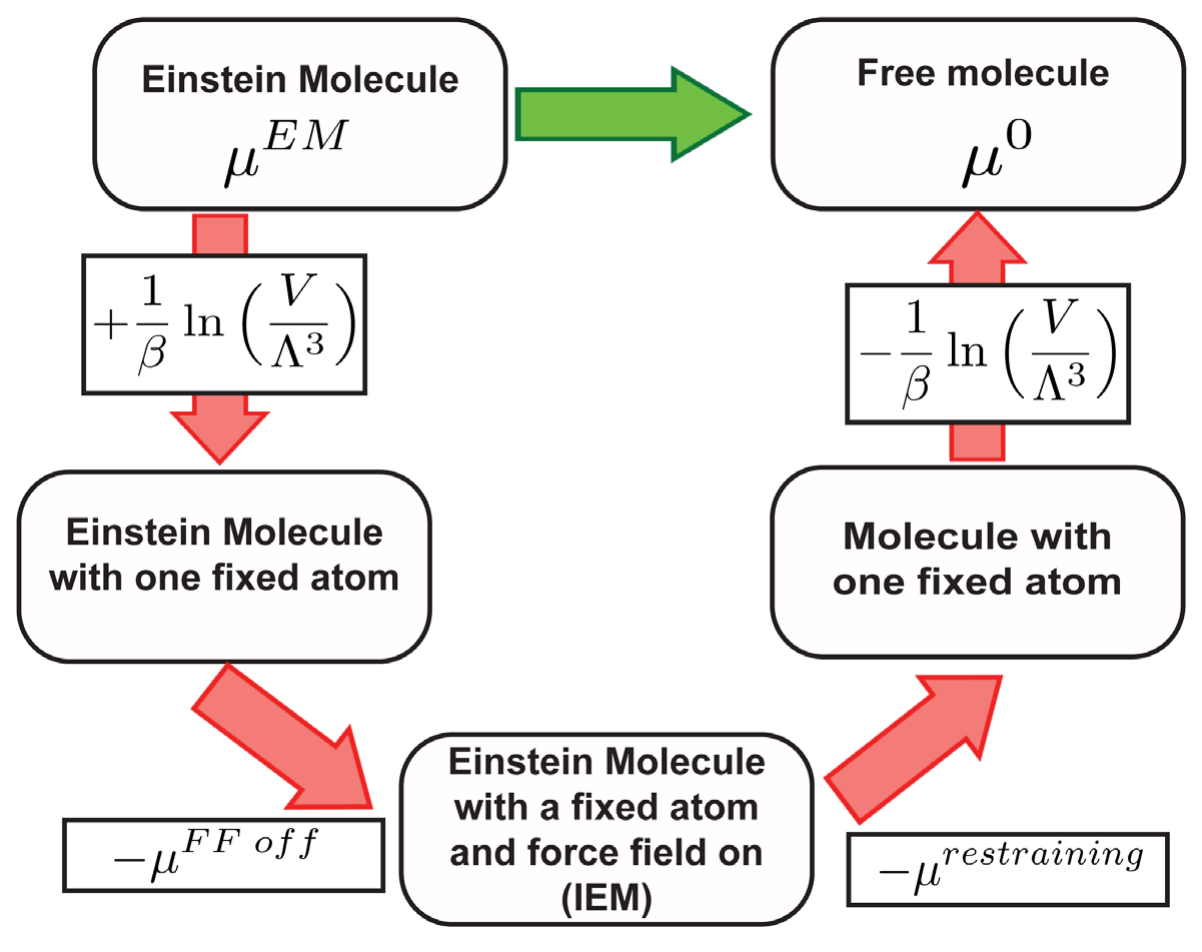
Thermodynamic cycle used to calculate the standard chemical potential of a molecule. Notice its similarity to Figure 1b.

In ECM, the free energy is calculated by:

$$A^{solid}=A_{FCM}^{EC}+\Delta A_{EC\to IEC}+\Delta A_{IEC\to SFCM}+\Delta A_{release\;CM}\;(8)$$

where $A_{FCM}^{EC}$ is the free energy of the Einstein crystal (EC) with a fixed center of mass (FCM); Δ *A*_*EC*→ *IEC*_ is the free energy difference between the Einstein crystal (EC) and the interacting Einstein crystal (IEC), i.e., the free energy difference in a transformation where the force field is progressively turned on throughout the calculation path. Δ *A*_*IEC*→ *SFCM*_ is the free energy difference between the IEC and the solid with a fixed center of mass (SFCM), i.e, turning off the harmonic restraints; and Δ *A*_*release CM*_ is the free energy of release of the center of mass (CM).

ECM can be difficult to implement because of the need for a fixed center of mass, so our work here is based on an alternative approach that is easier to implement. When particles move in ECM, the lattice needs to be moved because the center of mass is fixed ^36– 38^. Our method of choice, the Einstein Molecule Method (EMM, see Figure 1(b)), fixes a single atom in the lattice instead of the center of mass and is more easily implemented than ECM because of the relative difficulty of introducing center of mass restraints into existing simulation packages ^22, 37– 40^. EMM has been used to predict phase diagrams of TIP4P and SPC/E water models ^37^, free energies of ice polymorphs, solid methanol and toy systems ^40, 52^, and the solubilities of potassium and sodium chlorides ^22, 39^.

In EMM, the free energy of a solid is:

$$A^{solid}=A^{EM}+\Delta A_{EM\to IEM}+\Delta A_{IEM\to solid}\;(9)$$

where *A* ^*EM*^ is the free energy of the ideal Einstein molecule; Δ *A*_*id*→ *IEM*_ is the free energy difference between the ideal Einstein molecule and the interacting Einstein molecule (i.e, turning on the force field); and Δ *A*_*IEM*→ *solid*_ is the free energy difference between the interacting Einstein molecule and the solid (i.e, turning off the harmonic restraints). The advantage of EMM over ECM is the absence of the need to calculate a free energy term associated with releasing the fixed reference point ^37^.

Here, as per Equation 9, we compute the free energy of the solid by combining the absolute free energy of the ideal Einstein molecule with two terms that we calculate via alchemical free energy calculations—Δ *A*_*EM*→ *IEM*_ and Δ *A*_*IEM*→ *solid*_; these involve alchemically changing the interactions in the system. Numerical integration of Equation 10 allows the calculation of the ideal term, *A* ^*EM*^ ^40^:

$$A^{EM}=-\frac{1}{\beta }\ln Q_{EM}=\frac{1}{\beta }\ln\frac{N\Lambda^{3}}{V}-\frac{1}{\beta }\ln\int e^{-\beta U_{EM,1}(\Omega_{1})d\Omega_{1}}-\frac{\left(N-1\right)}{\beta }\ln\int \frac{1}{\Lambda^{3}}e^{-\beta U_{EM,2}(r_{2},\Omega_{2})dr_{2}d\Omega_{2}}\;(10)$$

where *A* ^*EM*^ and *Q*_*EM*_ are the free energy of the Einstein molecule and its partition function; *U*_*EM,*1_(Ω _1_) is the potential energy of the fixed particle 1; *U*_*EM,*2_( *r*_2_,Ω _2_) is the potential energy of a non-fixed particle at a distance *r*_2_ of particle 1; Ω _1_ and Ω _2_ are all the possible orientations the molecules can have in the lattice; Λ, *V*, *N*, and *β* respectively are the de Broglie wavelength, the system’s volume, its number of particles, and the reciprocal of *k*_*B*_ *T*, the product of the Boltzmann constant and the absolute temperature.

### The chemical potential of a component of a solution can be calculated using free energy calculations

Another critical component of computing the solubility of a compound is estimating the chemical potential of a solute in solution, since the solubility point is the concentration at which the chemical potentials of compound in the two phases are equal.

The chemical potential of a component *i* in solution, *µ*_*i*_, has an ideal and and excess component:

$\mu_{i}=-\frac{1}{\beta }\ln q_{i}+\frac{1}{\beta }\ln\frac{\Lambda_{i}^{3}N_{i}}{V}-\frac{1}{\beta }\ln{\langle e^{-\beta [U(N_{i}+1)-U(N_{i})]}\rangle }_{initial}\;(11)$

where *q*_*i*_ is the internal partition function of a single molecule of the solute, *U*( *N*_*i*_) is the potential energy of the system with *N*_*i*_ particles, Λ is the de Broglie thermal wavelength, and *V* is the system’s volume ^53^. 〈〉 _*initial*_ means that the term was obtained from an ensemble average over the configurations from the simulation of the initial state (see Equation 5). The first two terms of the equation above correspond to the ideal component of *µ*_*i*_; the last one, $\mu_{i}^{ex}$, corresponds to the excess component of *µ*_*i*_, and is associated with all non-ideal interactions of the extra component *i* with the solution (i.e. physical interactions that differ from those given by the ideal gas law). We obtained excess chemical potentials from solvation free energy calculations; the solute molecule is inserted in the solution by progressively turning on its interactions with the surrounding environment ^24, 28, 54^.

The challenge associated with the calculation of *µ*_*i*_ is the calculation of the standard chemical potential of *i*, $\mu_{i}^{0}$, the first term of Equation 11. *q*_*i*_, the internal partition function, includes the rotation, vibrational, electronic and nuclear partition functions of a single molecule ^53^ and is unknown. Here, we found a way of calculating $\mu_{i}^{0}$ without the knowledge of *q*_*i*_ by alchemically transforming a single solute molecule into a single Einstein molecule, whose absolute free energy we know how to calculate ^37, 38, 52^.

### Distinctives of this work

We are aware of three main approaches to compute the solubility of solids in solution using physical approaches: ECM-based methods ^21, 23^, EMM-based methods ^22, 39, 55^, and the approach of Michael Schnieders and collaborators which computes sublimation and solvation free energies and uses these in an alternate thermodynamic cycle to obtain solubility estimates ^15, 56^.

Many of the applications of these approaches have been to the solubility of ionic solids, with both ECM- ^21^ and EMM-based approaches ^22, 39, 55^ having some success. However, molecular solids introduce substantial additional complexities for both of these approaches.

The ECM *has* seen an initial test on solubility estimation. Li *et al.* ^23^ used the ECM to estimate the solubility of napthalene, but made several approximations such as assuming that the internal partition function component of the solute cancels between environments (perhaps justified given napthalene’s low solubility).

We are not aware of any work applying the EMM to solubility estimation of molecular solids; to our knowledge our work is the first to make such an attempt, though EMM has been used before to estimate the free energy of simple molecular solids ^40, 52^ but not the solubility. This explains our need to find our own approach to estimate $\mu_{i}^{0}$ for a single solute molecule.

A further distinctive of this work may be its treatment of solute flexibility within the ECM or EMM frameworks. Specifically, earlier work with EMM kept solutes rigid ^40, 52^, whereas the present work uses flexible solutes. It is worth noting, however, that the present solutes are still not especially flexible; acetylsalicylic acid is relatively rigid. While in principle the approach can handle flexible molecules, slow solute internal degrees of freedom will introduce additional sampling challenges. Since our focus here was on testing the general framework, we here chose to test on ASA, a relatively non-flexible molecule that allows us to avoid most issues with solute conformational sampling. It is likely that EMM would face additional challenges if applied to molecules with slow internal degrees of freedom or extensive flexible regions.

The Schneiders approach is an orthogonal one that we do not examine here.

## Methods

### Systems under study

Here, we chose three systems to study: An argon crystal for some small initial tests, *α*-methanol to help establish our protocol, and acetylsalicylic acid (ASA) as our main object of study. ASA is a known anti-inflammatory whose most stable polymorph, form I ^57^, has an aqueous solubility of approximately 0.038% mole fraction at 298 K ^58^. We also used *α*-methanol at 150 K and a toy face-centered cubic (fcc) argon crystal ^59^ to help us find an optimal protocol to calculate the absolute free energy of a molecular solid. *α*-methanol was chosen because it had been used before in a study that applied the EMM to calculate the absolute free energy of the solid ^40^.

All simulations were run in GROMACS 4.6.7 ^60– 63^. With one exception, all simulations used the General Amber force field (GAFF) version 1.7 with AM1-BCC charges ^64, 65^; the exception was *α*-methanol, because we ran these simulations using the input files – coordinates and force field parameters – provided by Aragonès *et al.*, who used an united atom version of the OPLS force field ^40^.

We simulated all solids and liquids using 5 ns Langevin dynamics simulations. ASA, *α*-methanol, and argon were simulated at 298.15 K, 150.0 K, and 4.0 K, respectively. Since water freezes at 273.15 K and we were not interested in the solubility of argon and methanol, there was no need to simulate aqueous solutions for these systems. Our simulations had the same length as the simulations run by Aragonès *et al.* All solid state simulations were run in NVT conditions. Liquid state simulations were run in NPT conditions; pressure was kept constant at 101.335 kPa using the Parrinello-Rahman barostat ^66^. We used the TIP3P water model ^67^ for all our liquid state simulations. More simulation details and example input files with full details can be found in the Supporting Information.

### Calculation of the absolute free energy of molecular crystals

The absolute free energies of the solids were calculated from trajectories of simulation boxes with 64 ASA molecules, 100 OPLS methanol molecules, and 864 argon atoms with periodic boundary conditions. ASA’s unit cell was obtained from Mercury CSD 3.8 ^68^ and the fcc argon crystal was obtained from the literature ^59^. Simulation box sizes were chosen to be approximately between 2 nm and 3 nm to ensure that box sizes were large enough that atoms and their periodic copies were not within cut-off distance of one another. *α*-methanol’s crystal was obtained from the Supporting information of Aragonès *et al.* ^40^ We used Amber14’s ambertools ^69– 72^ and ParmEd ^73^ to generate the ASA’s and argon’s solid state input files. All atoms but one were subjected to harmonic restraints in the x, y, and z coordinates.

A single atom was kept fixed in space to act as the reference point for the calculations, as explained in the Introduction. The choice of reference atom is in principle arbitrary. For ASA, here, we chose one of the carbon atoms in the aromatic ring. It is not uncommon in free energy calculations of various types, including binding free energy calculations ^74, 75^, to have to make arbitrary choices about which atoms to restrain, and several studies have demonstrated that such choices in practice are unimportant ^74, 75^. Thus, here, we were content to pick a single reference atom and not explore the impact this choice might have on convergence of the calculations, as there was no reason to expect this choice would have a significant impact on our calculations and the choice is unimportant for sufficiently long simulations.

Since the method does not include an angular-dependent orientational field and the harmonic restraints generate a considerable increase in energy when the position of two identical atoms are exchanged, our final results also include a simple analytical correction of − *N* ln (Σ _*rot*_)/ *β*, where Σ _*rot*_ is the number of proper rotations of the molecule ^40^.

Monte Carlo integration yielded *A*_*EM*_, the free energy of the Einstein molecule, as it was previously done for *α*-methanol in the literature ^40^. Δ *A*_*id*→ *IEM*_ and Δ *A*_*IEM*→ *solid*_ were estimated using TI ^44^ and the multistate Bennett acceptance ratio (MBAR) ^76^. We used force constants of 4000 k _B_T/Å ^2^ to restrain atoms to their lattice positions in ASA and argon simulations because it allowed us to use a reasonable time step of 1.0 fs in all simulations. α-methanol simulations used the same force constant that had been previously used by Aragonès *et al*. ^40^.

We used alchemical free energy calculations to obtain the difference in free energy between the reference Einstein molecule and the solid. This step was divided in two parts: (a) the force field parameters are alchemically turned on, and (b) the harmonic constraints are turned off.

Here, we deviate from earlier work which calculated the absolute free energy of a solid using EMM by introducing additional intermediate states to improve accuracy, along with using a superior free energy estimator.

For the calculation of Δ *A*_*id*→ *IEM*_, we found it was crucial to introduce intermediate states; we also switched to using the MBAR estimator. The original EMM calculation of the absolute free energy of a solid ^22, 37– 40, 52^ estimated Δ *A*_*id*→ *IEM*_ using exponential averaging (EXP) with just two states: the Einstein molecule (EM) and the interacting Einstein molecule (IEM) ^21, 22, 37– 40, 52, 55^. As EXP is known to have convergence issues and biases ^43, 45, 46, 50^, we switched to the superior MBAR free energy estimator ^76^. Additionally, when we did so, we found that overlap of states (as measured by the overlap matrix ^77^) was insufficient so we created a series of intermediate states connecting both ends of the transformation.

For Δ *A*_*IEM*→ *solid*_., the original work used TI ^44^. Here, we replaced TI with MBAR as our analysis method of choice. Generally, the literature shows that TI performs as well as more efficient methods like BAR and MBAR when the integrand is smooth ^43, 45, 46^, but it is sensitive to the choice and number of intermediate states ^78^. MBAR is the most consistently well-performing free energy estimator ^47^ and exploits the overlap between states more thoroughly than its predecessor, the Bennett Acceptance Ratio (BAR) estimator ^76^. Here, we chose to compare performance of MBAR and TI for calculation of Δ *A*_*IEM*→ *solid*_ for ASA and *α*-methanol; we also applied EXP as a comparison in the latter case only.

### Chemical potential calculations

The chemical potential of a pure solid is its molar free energy:

$$\mu =\frac{A}{N}\;(12)$$

where *N* is the number of molecules in the solid, and *A* its Helmholtz free energy.

The chemical potential of a substance *i* in water is defined as the derivative of the free energy of the system with respect to the composition:

$$\mu_{i}={\left(\frac{\partial G}{\partial N_{i}}\right)}_{P,T,N_{H_{2}O}}\;(13)$$

where *G* is the Gibbs free energy, and *N _i_* is the number of molecules of *i* in solution; *P*, *T*, and *N _H _2_O_* are the pressure, absolute temperature, and number of water molecules in solution, and are kept constant in the calculation.

One important aspect to discuss is the reason why we chose to calculate the Helmholtz free energy for the solid and Gibbs free energies for each solution. Solid state simulations with position restraints required running under constant temperature and constant volume conditions due to software limitations, therefore we were able to calculate *A* for the solids. At constant pressure, both kinds of free energy are related by:

ΔG=ΔA+PΔV(14)

Since solids are much less susceptible to volume changes than liquids, it is reasonable to consider that *P*Δ *V* is negligible and Δ *G* ≈ Δ *A*. For instance, the difference in volume between the experimental ASA crystal structure and the simulation box after a constant pressure equilibration stage is 0.14 nm ^3^. The *P*Δ *V* term – i.e., the free energy difference discounting possible structure relaxation effects – would be much smaller than the simulation error.

As we explain in more detail in the Results section, successful absolute free energy calculations for molecular solids require a pathway involving a large number of alchemical intermediate states. The calculation of the absolute free energies of *α*-methanol at 150 K and ASA required 600 states. Our analysis code only read each *λ* value to the fourth decimal place, and states needed to be spaced more closely together as as the harmonic restraints are turned off (see Supporting Information), we decided to split each free energy calculation into sets of 100 states.

Liquid state simulation boxes were generated using the SolvationToolkit ^79^, a Python package that uses packmol ^80^, OpenMolTools (v0.6.7) ^81^ and OpenEye Python Toolkits ^82– 84^. Excess chemical potentials were obtained with the same solvation free energy protocol used in previous studies ^28^: Starting from a fully interacting system, we progressively decouple the interactions of a single solute molecule with the remaining of the system, which allows us to calculate the free energy difference between a solute molecule in vacuum and in solution (i.e., the solvation free energy).

We also used alchemical free energy calculations using a single Einstein molecule as a reference state to estimate the standard chemical potential of a substance, $\mu_{i}^{0}$:

$$\;\mu_{i}^{0}=\mu_{i}^{ideal}-(\mu_{i}^{FFoff}+\mu_{i}^{restraining})\;(15)$$

where $\mu_{i}^{\text{FFoff}}$ and $\mu_{i}^{\text{restraining}}$ respectively are the chemical potential associated with turning off the force field and chemical potential of restraining the atoms of the molecule to their lattice positions ( Figure 2). $\mu_{i}^{\text{ideal}}$ is calculated using the Monte Carlo integration procedure that we used to calculate *A ^EM^* to a single molecule.

## Results

### Chemical potential of molecular solids

The first step to predict aqueous solubilities with the aid of absolute free energy calculations was the assessment of the methodologies we chose to use. Since our method is the same one used by Aragonès *et al.* ^40^ and we wanted to be sure that we could reproduce previous results, we ran simulations for *α*-methanol at 150 K and estimated the free energies of solids using MBAR. Turning off the harmonic restraints was the challenging step. Our MBAR calculation of Δ *A*_*IEM*→ *solid*_ for *α*-methanol using 18 intermediate states yielded −18(3) k _B_T, while our TI result was −18.421(5) k _B_T and the literature result was −17.33(3) k _B_T using 17 states ^40^. The MBAR error was unusually high (3 k _B_T), which is usually a signal of overlap problems or other serious concerns.

MBAR is a free energy estimation method that minimizes the free energy variance and considers the overlap between a given state and all the others in the transformation path ^46^, which means that high uncertainties (±3 *k _B_T*) suggest the presence of problems in the transformation’s path. TI’s uncertainty estimates are much lower, but we believe that this is an artifact. Error analysis for TI simply does not work the same way and does not give insight into whether exploration of phase space is adequate, unlike MBAR. Specifically, uncertainty estimates from TI usually factor in only the uncertainty in the integrand at each sampled lambda value and could potentially also factor in the smoothness of the integrand (i.e. numerical integration error) but do nothing to factor in whether the integrand will in fact vary smoothly in between lambda points; usually no data is available on this. BAR and MBAR, in contrast, factor in information about how well the intermediate states overlap in phase space and reflect high uncertainties when phase space overlap is poor. In our experience, TI would usually suffer from similar problems if additional intermediate states were added, but uncertainties in TI typically do not reflect this, as is the case here. Thus, the high uncertainty of the MBAR value indicates a sampling/convergence problem which warrants further exploration.

**Figure 3.**
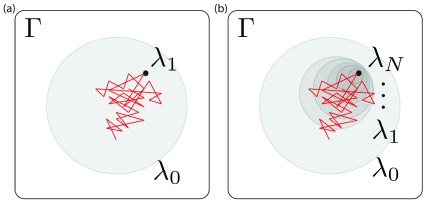
Phase space overlap between the states in a thermodynamic path for removing restraints with *λ*. Γ represents the phase space that contains all the configurations for all the states in the path. *λ*_0_ and *λ*_1_ (left) or *λ _N_* (right) represent the end states along the path, each shaded region represents a state in phase space and the red lines represent the configurations visited by the simulation run in the *λ*_0_ state. The restrained state is a subset of the unrestrained one. ( **a**) and ( **b**) represent simulations with different numbers of intermediate states along the path between a fully restrained state ( *λ*_1_ ( **a**) or *λ _N_* ( **b**)) and an unrestrained state ( *λ*_0_). In ( **a**), the simulation (red) only visits very few configurations consistent with the restrained state – i.e, there is poor phase space overlap – indicating a need for more intermediate states, otherwise any free energy estimates will be subject to very high uncertainties; in ( **b**) there is still almost no overlap between the simulation and states consistent with *λ _N_*, but there is overlap with the next shaded region, *λ*_1_, indicating the potential for overlap and accurate free energy estimates. Thus simulations run in each shaded region are more likely to have a bigger phase space overlap with *λ _N_* than simulations run in *λ*_0_.

**Figure 4.**
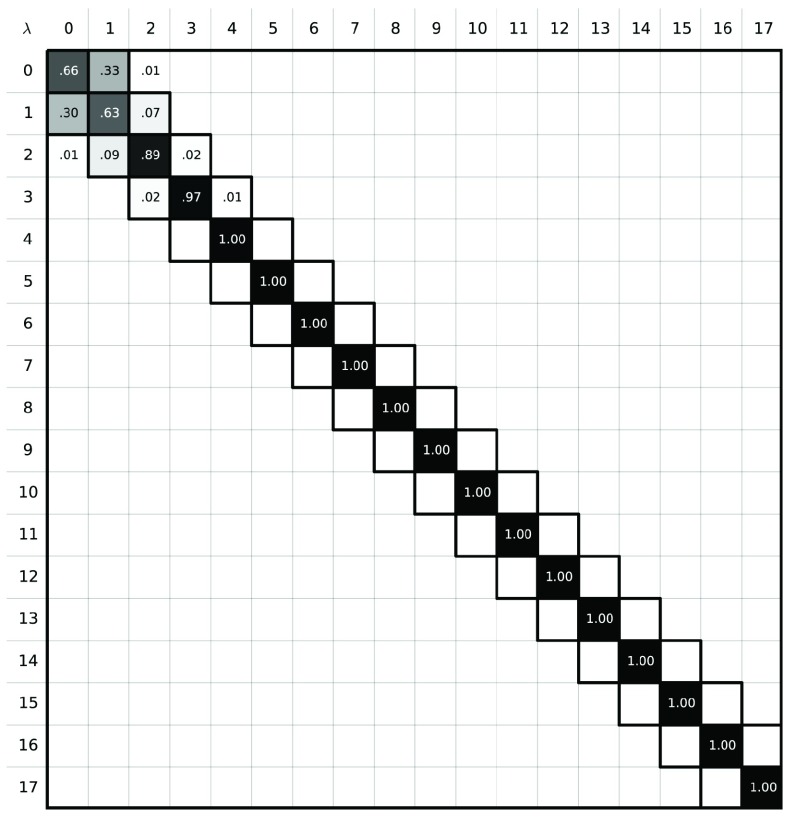
Phase space overlap between the states in the path between IEM and the *α*-methanol solid. The sum of all the elements in a row should yield 1.0, a probability of 100%. A good free energy estimate is obtained when the states along the alchemical path contain configurations that can be found in other intermediate states. In these situations, the phase space overlap is non-zero, which results in non-zero off-diagonal elements. Here, however, the phase space overlap plot shows that there is no overlap between the states *λ _i_*, 4 ≤ *i* ≤ 17 indicating poor free energy estimates will result.

To explore the high uncertainty of our MBAR free energy estimates, we examined the degree of overlap the intermediate states had with each other. Phase space overlap analysis ^85– 87^ quantifies the probability that any given configuration of an intermediate state can be found in other states. A good rule of thumb for designing a set of free energy calculations spanning between two states is to ensure that the states along the path have significant overlap with their neighbors as shown in Figure 3. More overlap improves the quality of the MBAR free energy estimation: Figure 3b represents a set of restraining simulations where the free energy uncertainty can potentially be accurately estimated using BAR and MBAR; Figure 3a shows a case where it cannot. In our case we find that the *α*-methanol simulation using 18 intermediate states does not have adequate overlap ( Figure 4)– specifically, the states 4 ≤ *λ _i_* ≤ 17 do not have overlapping configurations with other states, which explains the 3 k _B_T uncertainty in our MBAR estimate.

**Figure 5.**
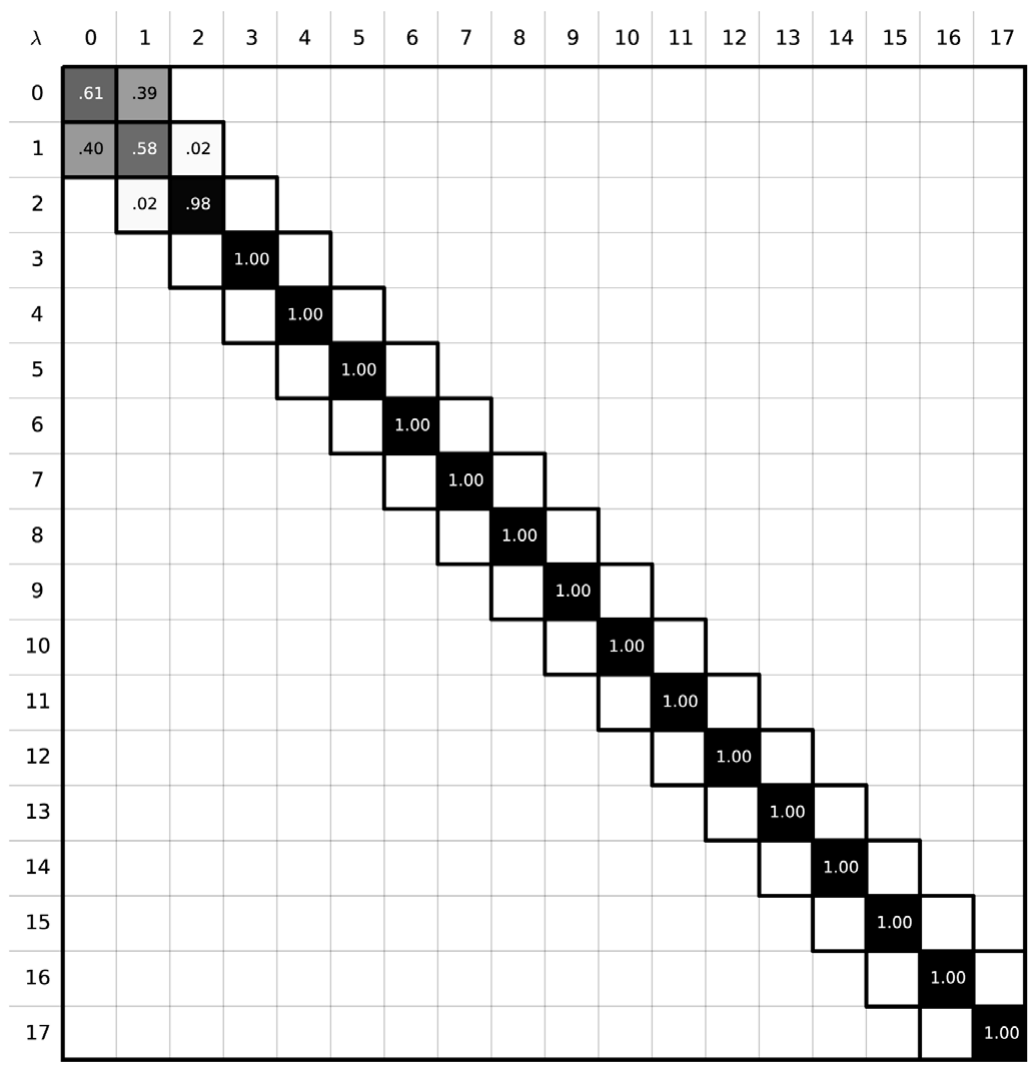
Phase space overlap between the states in the path between IEM and the fcc argon solid. A good free energy estimate is obtained when the states along the alchemical path contain configurations that can be found in other intermediate states. Here, however, the phase space overlap diagram shows that there is no overlap between the states *λ _i_*, 3 ≤ *i* ≤ 17, which explains the poor quality of the free energy result.

Since prior work had appeared to do this estimation successfully ^40^, we were uncertain why we were encountering such overlap problems, so we studied an even simpler system. We calculated Δ *A _IEM_*_→ *solid*_ of fcc argon at 4 K with 18 states as in our *α*-methanol free energy estimation. MBAR yielded an error estimate of infinity, whereas TI estimated Δ *A _IEM_*_→ *solid*_ to be −1666.5(8) k _B_T, which, as we show below, is incorrect. This path resulted phase space overlap diagram without overlap between the states after state number 2 ( Figure 5). Apparently as the harmonic potential that holds atoms in their lattice positions tends to zero, atoms become rather mobile, dramatically decreasing phase space overlap and leading to poor free energy estimates.

To improve phase space overlap, we introduced more intermediate states along the path for removing the restraints (see Figure 3). We chose to break down the simulation in smaller parts, adding a significant amount of states near the point where the harmonic restraints are approximately zero. The MBAR estimate of Δ *A _IEM_*_→ *solid*_ for fcc argon is −1016.0(2) k _B_T using 300 states. TI’s corresponding value was −1017(1) k _B_T, differing by far from the (incorrect) value of −1666.5(8) k _B_T obtained above with fewer states. Phase space overlap diagrams showed significant improvement in the configuration overlap between the states ( Supporting Information). Thus, increasing the number of states was an effective strategy, and we used it in all subsequent calculations.

Even though our *α*-methanol results were similar to results published previously by other authors ^40^, we need to emphasize that reliable free energies resulted from simulations with a large number of intermediate states, as can be seen in Table 1. Despite its conceptual simplicity, calculating the components of the absolute free energy of a solid to a point where there is significant phase space overlap between the intermediate states is computationally demanding. A 900-atom OPLS *α*-methanol system required 40 states to calculate Δ *A _id_*_→ *IEM*_, and 600 states for Δ *A _IEM_*_→ *solid*_. While this number of *λ* values gave sufficient overlap, we spent little effort optimizing it so substantial optimization may be possible, as we discuss below.

**Table 1.** Absolute free energy components for *α*-methanol at 150 K, in *k*_*B*_ *T*.

	Literature ^40^	Our replica
*A*_*EM*_	29.05	29.24(9)
Δ *A*_*id*→ *IEM*_	−41.27(1)	−38.04(7) (EXP) −41.306 56(4) (MBAR, 20 states) −41.275 719(7) (MBAR, 40 states)
Δ *A*_*IEM*→ *solid*_	−17.33(3)	−18.421(5) (TI, 18 states) −18(3) (MBAR, 18 states) −17.1712(6) (TI 600 states) −17.1692(4) (MBAR, 600 states)

We chose these intermediate states in advance, and these ultimately led to free energy errors smaller than 0.1 k _B_T; the estimated TI and MBAR values differed by no more than 0.3 k _B_T. Our results for ASA using an optimal number of states can be seen in Table 2. The MBAR chemical potential of ASA at 298.15 K equals to −221(3) k _B_T.

**Table 2.** Absolute free energy components for polymorph I of acetylsalicylic acid (ASA) at 298.15 K, in *k*_*B*_ *T*.

	Acetylsalicylic Acid
*A*_*EM*_	48(3)
Δ *A*_*id*→ *IEM*_	−167.316(1) (TI, 118 states) −167.07(3) (MBAR, 118 states)
Δ *A*_*IEM*→ *solid*_	−101.656(2) (TI, 600 states) −101.644(2) (MBAR, 600 states)

The uncertainty in the free energy for the ideal Einstein Molecule term is quite high (3 *k _B_T*). This could be improved via more careful Monte Carlo integration. Specifically, the Monte Carlo integrator of Equation 10 requires considerable tuning of numerical parameters for orientational change. Here, we chose a single set of parameters to use for both ASA and methanol simulations, which may not have been optimal, and resulted in a higher uncertainty in *A ^EM^* than presumably could have been achieved by more careful tuning for each individual case.

The computational cost of calculating *A ^ASA^* was high; Each state required a separate simulation (of a 1344-atom ASA system), with 718 states in total. Simulations typically required 11 hours on a single CPU, so the calculation of a single absolute free energy of a molecular solid required approximately 7898 CPU-hours.

It is worth noting that, in this proof of principle study, we devoted little effort to optimizing *λ* spacing, but considerable optimization might be possible. Specifically, restraint addition required a particularly large number of lambda values, but potentially this could be reduced considerably using cubically- or quartically-spaced lambda values as in related earlier work ^88^, potentially signifnicantly improving overlap while using far fewer intermediate states. This could reduce computational costs considerably. Additionally, the EMM approach requires the use of strong restraints, but we did not optimize the precise value of the restraining force constant; concievably, weaker restraints might also be acceptable, which would reduce the number of simulations needed for restraining and thus, corresponding, computational costs.

### Chemical potential of solutions and the solubility of GAFF ASA in TIP3P water

Equation 11 states that the absolute chemical potential of a solution is determined by three quantities: $\mu_{i}^{0}$, the standard chemical potential; $\mu_{i}^{ex}$, the excess chemical potential of the component at a concentration of *χ*; and a volume-dependent ideal gas component of *k _B_T* × ln ( $\Lambda_{i}^{3}\cdot$ *N _ASA_*/〈 *V*〉 *_solution_*). Calculation of $\mu_{ASA}^{0}$ only required information regarding the internal structure of the molecule ^53^, thus we estimated $\mu_{ASA}^{0}$ by alchemically transforming a single solute molecule into a single Einstein molecule ( Table 3), whose absolute free energy we know how to calculate. We used the same number of states that we chose for the solid state simulations and we found that $\mu_{ASA}^{0}$ is equal to –150.7(2) k _B_T, as discussed in the last subsection of the Methods section.

**Table 3.** Standard chemical potential of acetylsalicylic acid (ASA) at 298.15 K, in *k*_*B*_ *T*.

	Acetylsalicylic Acid
$\mu_{ASA}^{EM}$	9.3
$\mu_{ASA}^{FF\;off}$	65.7409(9) (MBAR 118 states)
$\mu_{ASA}^{restraining}$	94.3(2) (MBAR 600 states)

Concentrations, volumes and excess chemical potentials can be seen in Table 4. We obtained the excess chemical potentials from solvation free energy calculations ^24, 28, 54^. Volumes were obtained from the state in the alchemical path where the solute was fully coupled to the rest of the system.

**Table 4.** Simulation data for solutions of acetylsalicylic acid in water in different concentrations.

Molar fraction (%)	Volume ( *nm* ^3^)	# solute molecules	# solvent molecules	*µ ^ex^* ( *k _B_T*)
2.000 e-03	3035.99(5)	2	99998	−16.80(5)
6.666 e-03	911.17(2)	2	30002	−15.88(4)
7.999 e-03	759.33(1)	2	25000	−15.51(5)
9.998 e-03	911.45(3)	3	30003	−15.65(4)
9.999 e-03	607.59(2)	2	20000	−15.47(5)
1.3330 e-02	911.72(2)	4	30004	−15.77(4)
1.3332 e-02	455.84(2)	2	15000	−15.61(4)
1.666 e-02	912.00(3)	5	30005	−15.96(5)
1.9992 e-02	912.27(2)	6	30006	−15.78(4)
1.9996 e-02	304.01(1)	2	10000	−15.62(5)
3.998 e-02	152.25(1)	2	5000	−15.41(6)
1.996 e-01	30.835(7)	2	1000	−16.37(5)
2.991 e-01	31.069(3)	3	1000	−16.40(6)
3.984 e-01	31.309(7)	4	1000	−16.62(6)
4.975 e-01	31.547(3)	5	1000	−17.1(1)

**Figure 6.**
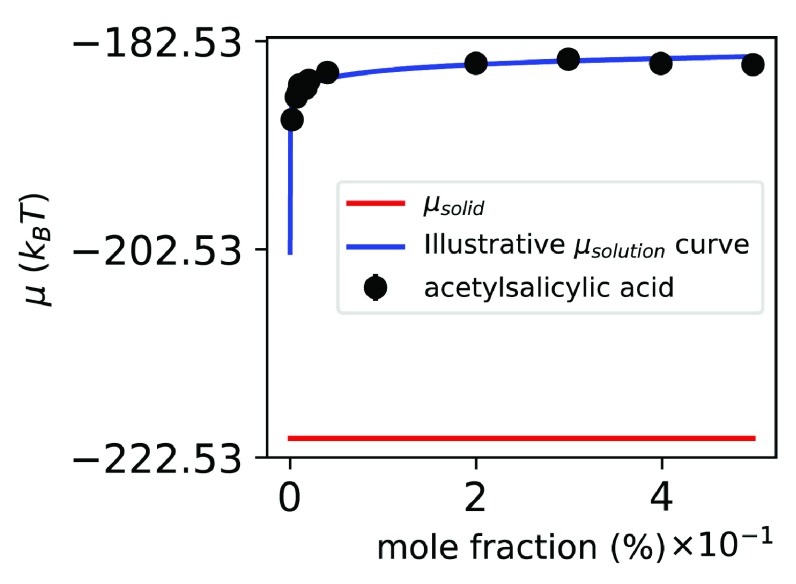
Chemical potentials of ASA, solid and solution in different concentrations, with respect to mole fraction.

The experimental aqueous solubility of ASA is approximately 0.038% in water at 298 K ^58^, but our model predicts that ASA is effectively insoluble in water ( Figure 6). While all-atom simulations can yield solubility estimates given adequate simulation time and a correct method, the computed solubility will be that dictated by the underlying energy model or force field, and will not necessarily match experiment. Here, we use GAFF, a general-purpose force field with known limitations ^28, 71, 89, 90^; apparently, here, the right answer **for the force field** is not correct. Perhaps this is because of limitations in describing the solid state, as the force field is parameterized for liquid state simulations. Indeed, classical fixed charge force fields have shown severe limitations for polymorph prediction for these reasons ^5, 31, 33– 35^. Also, point partial atomic charges regularly used in molecular dynamics do not describe electrostatic interactions in a solid particularly well ^91^. In the case of the ASA crystal, it is possible that its hydrogen bonds and *π*-stacking interactions add layers of complexity that are not properly described by GAFF.

## Discussion

Despite its theoretical rigor, solubility prediction from absolute free energy calculations is a difficult task: it is computationally expensive and, at least in the present approach, requires many different steps and a great deal of care. Here, we attempted to develop and test a general approach to compute the solubility of molecular solids by adapting the EMM to tackle this problem, as discussed above. Particularly, we were able to extend the EMM to calculation of the aqueuous solubility of molecular solids, and several of our modifications (such as the analysis technique employed and the number of intermediate states used) appear to make the calculations considerably more robust and precise.

To tune our methodology, we initially decided to reproduce the absolute free energy of solid *α*-methanol, one of methanol’s polymorphs, at 150 K using EMM before doing the same calculations for our compound of choice, ASA. We verified that the free energy differences between the Einstein molecule and the interactive Einstein molecule (Δ *A _EM_*_→ *IEM*_) and between the latter state and the solid (Δ *A _IEM_*_→ *solid*_) were more reliably estimated with the MBAR. The absolute free energy of the crystal (as computed for united-atom OPLS *α*-methanol) agreed with results found in the literature, which suggested that we were on the right path. We did, however, require a very large number of intermediate alchemical states to obtain accurate free energy estimates, making these simulations fairly computationally demanding.

We then chose to calculate the solubility of ASA, owing to its pharmacological importance and its relative complexity compared to previous molecular solids, whose absolute free energies have been computed via EMM previously ^40^. As for *α*-methanol, this calculation required a large number of intermediate alchemical states and considerable computational cost – approximately 8000 CPU hours for a single absolute free energy calculation for the molecular solid, even with the crystal structure as input. It seems likely the number of intermediate states could be further optimized, reducing costs, but clearly a large number of intermediate simulations was required and thus considerable computational cost. Despite all of this, we still could not reproduce the experimental aqueous solubility of ASA; experimentally it is modestly soluble, whereas our work would suggest it is essentially completely insoluble in water, likely due to force field limitations.

The solubility of naphthalene was recently estimated using a similar methodology, the Extended Einstein Crystal Method ^23^, but with additional approximations. Specifically, since naphthalene molecules interact very weakly with each other in the crystal lattice and with water molecules in solution, the differences between the internal partition function of a naphthalene molecule in the solid and in the solution were assumed to be negligible. This allowed the authors to drop some complexities in treatment of the solution-phase part of the calculation. However, that approach is only suitable for compounds that are only very weakly interacting in solution and in the crystal. ASA, in contrast, is a molecule that interacts strongly with other ASA molecules in its crystal lattice and with water molecules in solution via hydrogen bonds. For instance, an important crystalline feature that is not necessarily present in solution is the dimer structure, with two ASA molecules bound together via hydrogen bonds between the carboxylic acid groups. Differences between the internal partition functions of the molecule in the solid $\left(q_{ASA}^{solid}\right)$ and in solution $\left(q_{ASA}^{solution}\right)$ would probably not be negligible in this scenario, thus a more general approach is needed for treatment of such cases. Our work here provides one attempt in that direction.

Overall, the present approach seems to have significant limitations – most notably that the computational expense is considerable, and the resulting estimated solubility is quite inaccurate. Perhaps both of these may be surmountable; GPU-based free energy calculations can be dramatically faster, potentially reducing an 8000 CPU-hour calculation to 80 GPU hours, which would amount to overnight on 8 GPUs, and perhaps this could be optimized via changes to simulation time and number of intermediate states (such as via using cubically- or quartically-spaced states for restraining calculations ^88^). And with better force fields, perhaps accuracy could be improved; the AMOEBA-based approach of Schnieders shows considerable promise ^15^. New fixed-charge force fields such as AMBER ff15ipq ^92^ and AMBER ff15fb ^93^ could also be worth considering before using more expensive approaches, though such force fields would need generalization to cover small molecules before being applied to solubility calculation.

Alternatively, other approaches may be of interest. Solubility has been predicted by simulations using pseudocritical paths (i.e., paths were molecular crystals are transformed in tractable Einstein crystal-like states between the ending states of the transformation ^88, 94– 96,^) and a single experimental reference point ^96^), and with the aid of a thermodynamic cycle formed by the molecular crystal, the molecule in vacuum, and the solvated molecule ^15^. Absolute free energy of solids and fluids have also been calculated starting from different reference states ^97, 98^, and using supercritical path simulations ^99^.

We believe the time has come for routine physical methods for estimation of solubility, even if improved force fields prove necessary before results have significant accuracy for application to biomolecular design problems.

## Data availability

All data underlying the results are available as part of the article and no additional source data are required.

## Supplementary material

**Supporting Information. These files include GROMACS 4.6.7 input parameters for the simulation and all associated MDP files.** Also included is a file containing the elements of the phase space overlap matrix of a Δ *A*_*EM*→ *IEM*_, estimated from an alchemical path of 118 states.
