# Supplementary material for: Challenges in the use of atomistic simulations to predict solubilities of drug-like molecules
Source: F1000Res. 2019 Jan 4;7:686. Originally published 2018 May 31. [Version 2] doi: 10.12688/f1000research.14960.2 (PMC6069752; doi:10.12688/f1000research.14960.2)
Supplement: Supplementary file 1 [file f1000research-7-19081-s0000.tgz › 362c3a5d-25fd-43e1-881a-c6194e93ed7c.zip!/Supporting information/Data~/Supporting_Information/SupportingInformation.pdf]

Supporting Information
Challenges of the Use of Atomistic Simulations to Predict Solubilities of Drug-like Molecules

Guilherme Duarte Ramos Matos[1] and David L. Mobley[2]

[1]*Department of Chemistry, University of California, Irvine*

[2]*Departments of Pharmaceutical Sciences and Chemistry, University of California, Irvine**

I. SIMULATION DETAILS

The following are GROMACS 4.6.7 simulation input parameters, as are the MDP files with full details which are deposited in the Supporting Information.

General information

- Friction coefficient = $\text{mass}_{particle}/\tau_t$, $\tau_t = 2.0\,ps$.
- Parrinello-Rahman barostat (when applicable): $\tau_p = 10\,ps$ and compressibility = $4.5 \cdot 10^{-5}\,bar^{-1}$.

Electrostatics (solid)

- PME cut-off: 1.0 nm.
- PME order: 4
- Fourier spacing = 0.10 nm
- we used the same parameters as the Aragonès et al in solid state simulations. Additional details can be found in the MDP files deposited with this paper.

Electrostatics (solution)

- PME cut-off: 1.2 nm.
- PME order: 6
- Fourier spacing = 0.10 nm
- we used the same parameters as the Aragonès et al. for solid state simulations. Additional details can be found in the MDP files deposited with this paper.

vdW interactions

- Cut-off: $1.0\,nm$
- Switch at $0.9\,nm$
- DispCorr = AllEnerPres
- additional details can be found in the MDP files deposited with this paper.

Solution simulation files were generated using the `SolvationToolkit` module found at `https://github.com/MobleyLab/SolvationToolkit`. Solvation Toolkit relies on `openmoltools`, `mdtraj`, `packmol`, `ParmEd`, and OpenEye tools. As noted in the main body of the text, AM1-BCC charges were assigned with OpenEye's quacpac python module; we used `openmoltools` to drive this process. Specific source code used for charging is available at `https://github.com/choderalab/openmoltools/blob/v0.6.7/openmoltools/openeye.py#L13`. The code generates molecular conformations prior to charging, as was recommended at `http://docs.eyesopen.com/toolkits/cookbook/python/modeling/am1-bcc.html`.

We generated the simulation files for the acetylsalicylic solid simulations using the `pdb` file containing the crystal structure, Antechamber and `ParmEd`. We used the Antechamer AM1-BCC procedure to generate the charges for the solid state simulations.

Free energy estimation was done using the `alchemical_analysis` script which can be found at `https://github.com/MobleyLab/alchemical-analysis`.

II. SUPPORTING DETAILS

The `DA_ideal_to_IEM.csv` file containing the elements of the phase space overlap matrix of a $\Delta A_{EM \to IEM}$ estimated from an alchemical path of 118 states can be found with this Supporting Information.

DISCLOSURE STATEMENT

DLM is a member of the Scientific Advisory Board for OpenEye Scientific Software. JDC is a member of the Scientific Advisory Board for Schrödinger, LLC.

* dmobley@mobleylab.org
