# Supplementary material for: Challenges in the use of atomistic simulations to predict solubilities of drug-like molecules
Source: F1000Res. 2019 Jan 4;7:686. Originally published 2018 May 31. [Version 2] doi: 10.12688/f1000research.14960.2 (PMC6069752; doi:10.12688/f1000research.14960.2)
Supplement: Peer review history

# Peer review: Referee response for version 1

**DOI:** 10.5256/f1000research.16287.r36225

**Published:** 2018-07-25

**Authors:** Eric C. Dybeck

**Affiliations:** 1 Pfizer, Groton, CT, USA

**Summary and Overall Impressions**

 This article seeks to explore the application (and challenges) of using atomistic simulations to compute the solubility of drug-like small molecules. The ability to predict the solubility of emerging drug candidates is indeed an important challenge for the pharmaceutical industry, as many drugs which come out of discovery are BCS class II or IV with low aqueous solubility. The method proposed in this work could in principle allow medicinal chemists and pharmaceutical scientists to evaluate drug solubility early in the development pipeline and accelerate product release. This method is sufficiently novel and well executed to deserve publication in this scientific journal. The authors have also done a fantastic job including the details and input files necessary for one well-versed in the art to reproduce their results. Minor revisions are suggested below to further improve the clarity and quality of the article.

**Suggested Revisions**

1. In the original papers by Aragones, Noya, and Vega, the molecules were constrained to be completely rigid. In this work, the investigators appear use this method for both constrained and fully flexible molecular systems. The authors should consider highlighting this expanded capability, and perhaps discuss the tradeoffs in accuracy and simulation speed between flexible vs rigid molecular treatment for absolute solubility prediction.
2. The authors use both the term ‘restraints’ and ‘constraints’ to describe the harmonic potential being applied and removed from atoms in the system. It may be more clear to consistently refer to these alchemical harmonic potentials as ‘restraints’ and reserve the term ‘constraint’ for the subroutine used to keep molecules fully rigid.
3. An important feature in the Einstein molecule method utilized herein is the use of a frozen reference atom rather than the traditional full-system center-of-mass removal. In principle, the free energy to add restraints to a system with a frozen atom will be independent of the choice of reference. In practice, some choices of reference atom may lead to faster simulation convergence than others due to differences in the fluctuation magnitude of the atoms around their natural lattice positions. It would be useful to discuss best practices in how one chose the frozen reference atom, as well as to discuss the effect of different reference choices on the convergence of the various alchemical steps in this workflow.
4. The authors mention using a previously developed Monte Carlo code to compute the absolute free energy of the reference Einstein Molecule state. It would be useful to comment on the uncertainty inherent in this component of the overall free energy calculation. For example, how much variance would ten independent calls to the Monte Carlo program have for these compounds?
5. On page 7 of the paper, the authors briefly mention that “GROMACS only reads each lambda value up to the 4 ^th^ decimal place”. In my own alchemical simulations with GROMACS, I have routinely used lambda values out to 8 decimal places. If this is a version-specific limitation, the authors should state this explicitly. Otherwise, this comment should be removed.
6. The investigators chose to use linear spacing for the lambda values in all alchemical processes including the addition of harmonic restraints to the physical system. They also note that adding harmonic restraints represented the most time-intensive part of the overall workflow and required splitting into 6 different steps of increasing restraint strength. Furthermore, they find that the overlap between neighboring lambda states during the restraint addition is quite low (Figure 2-4) and produces large uncertainties in their final free energy estimates. In my own investigations of adding harmonic restraints to solids from 2016 (cited in this work) I observed that either cubically- or quartically- spaced lambda values significantly improve the overlap along the thermodynamic path relative to linear spacing and reduce the total amount of simulation cost. This should be included as a potential remedy for the high simulation cost lamented in the discussion section of the paper.
7. The investigators chose to use a large value of >1,000,000 kJ/nm/mol for their final restraint state to add or remove inter-particle interactions. It is necessary to have strong restraints in order to remove stiff degrees of freedom such as bonds and angles. However, it is possible that the interaction removal could be achieved with a weaker value of the restraint constant, and this would in turn reduce the number of simulations to add or remove harmonic restraints. This should also be discussed in the context of ways to reduce the amount of simulation expense observed for these calculations.
8. Finally, the authors should consider including the additional papers of Sellers et al. 2016 ^1^ and Schilling and Schmid 2009 ^2^ who also explore the use of atomistic simulation to compute absolute solid free energies. These articles also discuss how to apply restraints in a manner that preserves the indistinguishability of certain particles. It would be worth discussing ways to account for particle indistinguishablility in the method presented in this paper.

I have read this submission. I believe that I have an appropriate level of expertise to confirm that it is of an acceptable scientific standard.

## References

[1] SellersMSLísalMBrennanJK: Free-energy calculations using classical molecular simulation: application to the determination of the melting point and chemical potential of a flexible RDX model. *Phys Chem Chem Phys*.2016;18(11) : 10.1039/c5cp06164d 7841-50 10.1039/c5cp06164d 26661376

[2] SchillingTSchmidF: Computing absolute free energies of disordered structures by molecular simulation. *J Chem Phys*.2009;131(23) : 10.1063/1.3274951 231102 10.1063/1.3274951 20025306

# Peer review: Referee response for version 1

**DOI:** 10.5256/f1000research.16287.r34597

**Published:** 2018-06-25

**Authors:** Lillian T. Chong, Anthony T. Bogetti

**Affiliations:** 1 Department of Chemistry, University of Pittsburgh, Pittsburgh, PA, USA

The authors report extensive computations of absolute chemical potentials to predict the solubility of the drug, acetylsalicylic acid (aspirin), using molecular dynamics simulations. The manuscript is clearly written, providing the relevant background for understanding their results, a non-trivial task for this subject matter. A non-expert in the field of statistical mechanics should have little trouble reading this paper due to such careful and effective writing. In addition, figures such as Figure 1 are well constructed and greatly aid in the understanding of the relevant theory. While the results are not ideal, the challenges and limitations that have been revealed are informative and important for moving forward in the field of drug discovery. This manuscript would be of broad interest to life scientists. I recommend indexing of this manuscript in *F1000Research*.

 I have only a few comments for minor revisions:

1. Introduction section: This section could be re-framed to accentuate the positive, informative aspects of this manuscript’s results. For instance, it would be worth mentioning the fact that the new thermodynamic cycle employed in this study was able to enhance solubility calculations of the methanol system, an important feature of this manuscript that should be highlighted early on. In addition, the example of Norvir in the first paragraph could be shortened considerably, and similar shortening of the Introduction could be more effective in presenting the broader impacts of this study.
2. Theory section: This section would benefit from a clear definition of an Einstein crystal for life scientists at the very beginning. Also important would be to include early in the Theory section explanations of the ECM and EMM cycles and logically structuring the rest of the section from there, including more fundamental equations as needed. Also, it is not clear in equation (5) what is meant by averaging over the configurations of the initial state. Please clarify. 
3. Distinctives of this Work section: It would be beneficial to remind the reader of the unique aspects of the EMM method over ECM since the implementation of this method is the novel in the manuscript.
4. Methods section, third paragraph: It was not clear to this reader which temperatures were used for the liquid simulations. Please clearly mention the temperatures. If the simulations were run at 4 K, then the authors should comment on the accuracy of the TIP3P water model at this very low temperature.
5. Discussion section, second to last paragraph: The authors mention that “with better force fields, perhaps accuracy could be improved.” Regarding “better force fields”, it would be worth mentioning two recent fixed-charge force fields, AMBER ff15ipq and AMBER ff15fb, that have been developed using sweeping optimizations of hundreds of parameters simultaneously using automated tools and could be worth considering before using more expensive polarizable force fields such as AMOEBA.

We have read this submission. We believe that we have an appropriate level of expertise to confirm that it is of an acceptable scientific standard.
